# Normalizing biomedical terms by minimizing ambiguity and variability

**DOI:** 10.1186/1471-2105-9-S3-S2

**Published:** 2008-04-11

**Authors:** Yoshimasa Tsuruoka, John McNaught, Sophia Ananiadou

**Affiliations:** 1 School of Computer Science, The University of Manchester, MIB, 131 Princess Street, Manchester, M1 7DN, UK; 2 National Centre for Text Mining (NaCTeM), MIB, 131 Princess Street, Manchester, M1 7DN, UK

## Abstract

**Background:**

One of the difficulties in mapping biomedical named entities, e.g. genes, proteins, chemicals and diseases, to their concept identifiers stems from the potential variability of the terms. Soft string matching is a possible solution to the problem, but its inherent heavy computational cost discourages its use when the dictionaries are large or when real time processing is required. A less computationally demanding approach is to normalize the terms by using heuristic rules, which enables us to look up a dictionary in a constant time regardless of its size. The development of good heuristic rules, however, requires extensive knowledge of the terminology in question and thus is the bottleneck of the normalization approach.

**Results:**

We present a novel framework for discovering a list of normalization rules from a dictionary in a fully automated manner. The rules are discovered in such a way that they minimize the ambiguity and variability of the terms in the dictionary. We evaluated our algorithm using two large dictionaries: a human gene/protein name dictionary built from BioThesaurus and a disease name dictionary built from UMLS.

**Conclusions:**

The experimental results showed that automatically discovered rules can perform comparably to carefully crafted heuristic rules in term mapping tasks, and the computational overhead of rule application is small enough that a very fast implementation is possible. This work will help improve the performance of term-concept mapping tasks in biomedical information extraction especially when good normalization heuristics for the target terminology are not fully known.

## Background

Named entities such as names of genes, proteins, chemicals, tissues, and diseases play a central role in information extraction from biomedical documents [1-3]. To fully utilize the information they convey in the document, we generally need to perform two steps. In the first step, which is commonly called named entity recognition, we identify the regions of text that are likely to be named entities and classify them into predefined categories. Substantial research efforts have been devoted to the improvement of named entity recognizers, and today we can identify biomedical named entities in the literature with reasonable (although still not entirely satisfactory) accuracy by using rule-based or machine learning-based techniques [4-8].

In the second step, we map the recognized entities with the corresponding concepts in the dictionary (or ontology). This step is crucial for making the extracted information exchangeable at the concept level [9]. This mapping task has proven to be non trivial especially in the biomedical domain [10-13]. One of the main problems is that biomedical terms have many potential variants, and it is not possible for a dictionary to cover all possible variants in advance.

One possible approach to tackle this problem is to use soft string matching techniques. Soft matching enables us to compute the degree of similarity between strings, and thus we can associate a term with its concept even when the dictionary fails to contain the exact spelling of the term. In fact, soft matching methods have been shown to be useful in several gene/protein name mapping tasks [11,13,14]. Soft string matching, however, is not without drawbacks: the method requires a considerable computational cost when looking up the dictionary [15]. This problem is particularly serious when we use large dictionaries such as those for gene/protein names and disease names, which can contain more than hundreds of thousands terms. Although there are techniques to speed up the computation for simple similarity measures like uniform-cost edit distance [16], it is hard to apply those techniques to the sophisticated similarity measures needed in real mapping tasks. To make matters worse, the size of the literature that we need to analyze for biomedical information extraction could be huge—MEDLINE abstracts contain more than 70 million sentences, let alone full papers.

Another approach to alleviate the problem of term variation is to normalize the terms by using heuristic rules [17-19]. For example, converting capital letters to lower case has been shown to be an effective normalization rule for gene/protein names [17]. The distinct advantage of the normalization approach over the soft matching approach is the speed of looking up the dictionary. Once the terms are normalized, we can use a *hashing* technique to lookup a dictionary with a constant computational cost regardless of its size, while the cost for soft matching increases linearly with the size of the dictionary.

What is most important in the normalization approach is how we normalize the terms. Bad heuristic rules often lose important information in the terms. For example, deleting *all* digits from a term is probably a bad rule for gene/protein names, because the rule makes it impossible to distinguish ‘ACE1’ and ‘ACE2’ although it enables us to match ‘ACE’ with ‘ACE1.’

Although using good heuristic rules is certainly important, their development is not straightforward. It requires good intuition and extensive knowledge of the terminology in question; the developer has to know the types of variation and potential side effects of normalization. Consequently, it remains to be seen what normalization rules would work well for various classes of named entities in the biomedical domain.

In this paper, we present a novel approach for the automatic discovery of term normalization rules, which requires no expert knowledge of the terminology. To achieve this goal, we leverage the important insight provided in previous studies [17,20] in which contrast and variability in gene names were analyzed to test the effectiveness of several normalization heuristics. Their work suggests that one could distinguish good normalization rules from bad ones by analyzing the effect of normalization on the relationships between terms and their concept IDs in the dictionary. We take their work one step further and present a framework for discovering a list of “good” normalization rules from a dictionary in a fully automated manner.

## Methods

### Ambiguity and variability

In this section, we describe two notions that are needed to quantify the utility of a normalization rule. We call them *ambiguity* and *variability*.

First, let us define a dictionary simply as a list of terms {*t*_1_, …, *t_N_*} where each term is associated with a concept ID *c_j_* ∈ {*c*_1_, …, *c_M_*}. In the biomedical domain, concept IDs typically correspond to the unique identifiers for conceptual entities such as genes, chemicals, and diseases defined in biomedical databases (e.g. UniProt, InChI, and OMIM).

Table 1 shows an imaginary dictionary consisting of only three concept IDs. Here, we define two values for the dictionary: the ambiguity value and the variability value. The ambiguity value quantifies how ambiguous, on average, the terms in the dictionary are. More specifically, we define the ambiguity value as follows:

$$\left(ambiguity\right)=\;\frac{1}{N}\;\sum_{i=1}^{N}C\left(t_{i}\right),$$

where *N* is the number of terms in the dictionary, and *C*(*t_i_*) is the number of the concept IDs that include a term whose spelling is identical to *t_i_*.

**Table 1:** Ambiguity and variability in a dictionary. This is an imaginary dictionary consisting of three concept IDs. All terms belonging to the same concept ID are assumed to be synonymous (conveying the same meaning).

Concept ID	Term
1	IL2
1	IL-2
1	Interleukin
2	IL3
2	IL-3
2	Interleukin
3	ZFP580
3	ZFP581
3	Zinc finger protein

The variability value, in contrast, quantifies how variable the terms are. This is calculated as:

$$\left(variability\right)=\;\frac{1}{M}\;\sum_{j=1}^{M}T\left(c_{j}\right),$$

Where *M* is the number of concept IDs in the dictionary, and *T*(*c_j_*) is the number of *unique* terms that the concept *c_j_* includes.

For the dictionary shown in Table 1, we can calculate these values as follows:

$$\begin{array}{c} \left(ambiguity\right)\;=\;\;\;\;\;\;\;\frac{1}{9}\;\left(1\;+\;1\;+\;2\;+\;1\;+\;1\;+\;2\;+\;1\;+\;1\;+\;1\right)\\ \;\;\;\;\;\;\;\;\;\;\;\;\;\;\;\;\;\;\;\;\;\;\;\;\;\;\;\;\;\;\;\;\;\;\;\;=\;\;\;\;\;\;1.22...\\ \left(variability\right)\;=\;\;\;\;\;\;\frac{1}{3}\;\left(3\;+\;3\;+\;3\right)\\ \;\;\;\;\;\;\;\;\;\;\;\;\;\;\;\;\;\;\;\;\;\;\;\;\;\;\;\;\;\;\;\;\;\;\;\;\;\;=\;\;\;\;\;3 \end{array}$$

These values can be seen as the indicators of the complexity of terminology. Ideally, we do not want the terms to be ambiguous or variable, because both lead to impaired performance in mapping tasks. We thus favour smaller values for these factors.

Now let us see how a normalization rule can change the situation. Suppose that we have the normalization rule that removes hyphens. By applying it to the terms in the dictionary, ‘IL-2’ becomes ‘IL2’, and ‘IL-3’ becomes ‘IL3’. Then we obtain new values for ambiguity and variability:

$$\begin{array}{c} \left(ambiguity\right)\;=\;\;\;\;\;\;\;\frac{1}{9}\;\left(1\;+\;1\;+\;2\;+\;1\;+\;1\;+\;2\;+\;1\;+\;1\;+\;1\right)\\ \;\;\;\;\;\;\;\;\;\;\;\;\;\;\;\;=\;\;\;\;\;\;1.22...\\ \left(variability\right)\;\;\;=\;\;\;\;\;\;\;\frac{1}{3}\;\left(2\;+\;2\;+\;3\right)\\ \;\;\;\;\;\;\;\;\;\;\;\;\;\;\;\;=\;\;\;\;2.33... \end{array}$$

We have succeeded in reducing the variability value without increasing the ambiguity. This indicates that this normalization rule is a good one.

For the same dictionary shown in Table 1, we could think of a different normalization rule that replaces all digits with the special symbol ‘#’. If we apply this rule to the dictionary, ‘IL2’ and ‘IL3’ become ‘IL #’, ‘IL-2’ and ‘IL-3’ become ‘IL-#’, and ‘ZFP580’ and ‘ZFP581’ become ‘ZFP###’. We then obtain:

$$\begin{array}{c} \left(ambiguity\right)\;=\;\;\;\;\;\;\;\frac{1}{9}\;\left(2\;+\;2\;+\;2\;+\;2\;+\;2\;+\;2\;+\;1\;+\;1\;+\;1\right)\\ \;\;\;\;\;\;\;\;\;\;\;\;\;\;\;\;=\;\;\;\;\;\;1.66...\\ \left(variability\right)\;\;\;=\;\;\;\;\;\;\;\frac{1}{3}\;\left(3\;+\;3\;+\;2\right)\\ \;\;\;\;\;\;\;\;\;\;\;\;\;\;\;\;=\;\;\;\;\;\;\;2.66... \end{array}$$

Although we have a decreased value for variability, the ambiguity value has increased. This indicates that this normalization rule may not be a good one.

The examples above demonstrate that we could distinguish good normalization rules from bad ones by observing the change of the ambiguity/variability values defined in the dictionary. In general, a normalization rule reduces the variability value at the sacrifice of the increase in the ambiguity value. Therefore, what we want is a rule that can maximize the reduction of the variability value and keep the increase of the ambiguity value minimal.

We now need to integrate the two values in order to quantify the overall “goodness” of a normalization rule. We define a new value, which we call *complexity*, as follows:

$$\left(complexity\right)\;\;\;\;=\;\;\;\;\;\left(ambiguity\right)\;\;\;\;\times \;\;\;\;{\left(variability\right)}^{\alpha },$$

where α is the constant that determines the trade-off between ambiguity and variability.

Now the problem has become very simple; if a normalization rule can reduce the complexity value for the dictionary, then the rule is a good rule, otherwise it is a bad rule.

### Generating rule candidates

The next problem is how we automatically generate normalization rules. Ideally, we want to allow normalization rules to be of any type, such as regular expressions and context-free grammars. However, we found it difficult to incorporate such complex rules in a fully automatic manner because it entails a huge search space for rule hypotheses.

In this work, we focus only on character-level replacement rules. By focusing on this type, we can easily generate rule candidates from the terms in the dictionary. For example, the first and the second terms in the dictionary given in Table 1 constitute the following pair.

IL2

IL-2

From this pair, we can easily see that we will be able to match the two terms if we remove the hyphens (i.e. replace the hyphens with the *null* character), which in turn will reduce the variability value of the dictionary. In other words, we can automatically generate the rule that removes hyphens, by observing this term pair.

More formally, we can represent any pair of terms X and Y as follows:

LX_C_R

LY_C_R

where L is the left common substring shared by X and Y, R is the right common substring, and X_C_ and Y_C_ are the substrings at the center that are not shared by the two strings. From this representation, we create the rule that replaces Y_C_ with X_C_, which will transform Y into *X*.

For the above example pair ‘IL2’ and ‘IL-2’, L is ‘IL’, R is ’2’, Y_C_ is ‘-’, and X_C_ is the null character. If we take the first term ‘IL2’ and the third term ‘Interleukin’ from the dictionary in Table 1, L is ‘I’, R is the null character, Y_C_ is ‘nterleukin’, and X_C_ is ‘L2’.

### Discovering rules

In the previous sections, we have defined a measure to quantify the utility of a normalization rule and presented a method to generate a rule candidate from any given term pair. Now we describe the whole process of rule discovery. The process is as follows:

1. Generate rule candidates from all possible pairs of synonymous terms in the dictionary (i.e. terms sharing the same concept ID).

2. Select a rule that can reduce the complexity value defined by Equation 3.

3. Apply the rule to all the terms in the dictionary.

4. Go back to 1—repeat until the predefined number of iterations is reached.

Notice that the process is iterative—we apply the discovered rule immediately to the dictionary and then use the updated dictionary for the next iteration. This is because the rules discovered are to be used in sequential manner; the end product of our rule discovery system is a list of normalization rules, and we shall use them exactly in the same order specified in the list. Thus the terms in the dictionary have to be sequentially updated in the rule discovery process to make sure that they go through the same rule applications.

In step 2, we need to select a good rule from the rule candidates generated in step 1. The obvious strategy would be to select the rule that maximizes the reduction of the complexity value of the dictionary. However, we found this strategy impractical when the dictionary is large, because it requires us to try applying every rule candidate to the dictionary to see its utility. In this work, we use a less computationally intensive strategy. First, we sort the rule candidates in descending order of frequency of occurrence. We then pick up the first rule that can decrease the complexity value. This strategy worked reasonably well, since the rule candidates that are generated many times decrease the variability value to a greater degree than infrequent ones do.

To further improve the efficiency of the entire process, we do not consider any rule candidates that have failed once to reduce the complexity value. This pruning method is not completely safe, because the terms in the dictionary change as the process proceeds and thus a candidate that has been rejected once could become acceptable at some point. However, we found that the speed-up gain outweighs the demerit when the dictionary is large.

## Results and discussion

### Dictionaries

We used two large-scale dictionaries for the experiments to evaluate our rule discovery algorithm. One is a dictionary for human gene/protein names, and the other for disease names.

The gene/protein name dictionary was created from BioThesaurus [21], which is a collection of more than two million gene/protein names for various species. We selected only the human genes/proteins by consulting the UniProt database [22] and removed the names that were nonsensical (e.g. IDs for other databases). The resulting dictionary consisted of 14,893 concept IDs and 205,909 terms.

The disease dictionary was created from UMLS Metathesaurus [23], which is a large multi-lingual vocabulary database that contains biomedical and health related concepts and their various names. We extracted all entries that were associated with the semantic type “Disease or Syndrome.” The resulting dictionary consisted of 48,391 concept IDs and 148,531 terms.

Table 2 shows statistics of the dictionaries. Note that the terms in the gene/protein dictionary are highly ambiguous in the first place. For evaluation purpose, we also created a reduced version for each dictionary by removing all ambiguous terms.

**Table 2:** Statistics of the dictionaries

Dictionary	#Concept IDs	#Terms	Ambiguity	Variability
Gene/protein name dictionary (original)	14,893	205,909	5.715	13.826
Gene/protein name dictionary (reduced)	14,882	174,162	1.000	11.703
Disease dictionary (original)	48,391	148,531	1.005	3.069
Disease dictionary (reduced)	48,391	147,859	1.000	3.056

### Evaluation using held-out terms

As discussed in the Methods section, we create normalization rules in such a way that they minimize the variability and ambiguity of the terms in the dictionary. We thus know that they are “good” rules for the terms included in the dictionary. It is, however, not clear if those rules are also appropriate for the terms that are not included in the dictionary. In other words, we need to evaluate how the discovered rules will help map *unseen* terms with their correct concept IDs.

One way of evaluating such performance is to use a *held-out* data set for evaluation. Before executing a rule discovery process, we remove some randomly selected terms from the dictionary and keep them as separate data. We then execute the rule discovery process. The mapping performance is then evaluated by applying the discovered rules also to the held-out terms and looking them up in the dictionary, where the lookup system produces, for each heldout term, zero or more concept IDs by exact string matching. The overall lookup performance can be evaluated in terms of precision and recall. Precision is given by

$$\left(precision\right)=\frac{n_{c}}{n_{m}},$$

where *n_m_* is the total number of concept IDs output by the lookup system, and *n_c_* is the total number of correct concept IDs output by the system. Recall is given by

$$\left(recall\right)\;=\;\frac{n_{c}}{n_{h}},$$

where *n_h_* is the number of heldout terms.

With these performance measures, we carried out several sets of experiments for automatic rule discovery. Throughout all experiments reported in this paper, we set the tradeoff parameter α in Equation 3 to 0.1. All capital letters in the terms are converted to lower case before applying our rule discovery algorithm.

Table 3 shows the result for the human gene/protein dictionary. We used the reduced version of the dictionary in this experiment in order to make clear how normalization affects the precision of lookup performance (i.e. the lookup precision without normalization is ensured to be 100%). We used 1,000 heldout terms for evaluation. The first column shows the iteration counts of the rule discovery process. The second column shows the values of ambiguity and variability in the dictionary. The third column shows the rules discovered. The fourth column shows the lookup performance evaluated using the heldout terms.

**Table 3:** Discovering rules from a gene/protein dictionary

	Dictionary			Lookup performance	
Iter.	Ambiguity	Variability	Rule	Precision	Recall

0	1.004	10.399	*(convert capital letters to lower case)*	0.975	0.194
1	1.006	10.101	‘ ’ → ‘-’	0.967	0.233
2	1.009	9.759	‘-’ → ‘’	0.966	0.280
3	1.012	9.318	‘protein’ → ‘’	0.958	0.340
4	1.013	9.155	‘precursor’ → ‘’	0.959	0.347
5	1.013	9.038	‘,’ → ‘’	0.961	0.366
6	1.013	9.006	‘incfinger’ → ‘nf’	0.961	0.368
7	1.013	8.979	‘isoforma’ → ‘’	0.962	0.375
8	1.013	8.953	‘isoformb’ → ‘’	0.962	0.377
9	1.013	8.937	‘prepro’ → ‘’	0.962	0.379
10	1.013	8.916	‘ike’ → ‘’	0.962	0.380
11	1.013	8.911	‘rotocadherin’ → ‘cdh’	0.962	0.380
12	1.013	8.891	‘(drosophila)’ → ‘’	0.962	0.383
13	1.013	8.873	‘variant’ → ‘’	0.962	0.384
14	1.014	8.867	‘nterleukin’ → ‘l’	0.962	0.384
15	1.014	8.857	‘drosophilahomologof’ → ‘homolog’	0.963	0.385
16	1.014	8.846	‘coupledrecepto’ → ‘p’	0.963	0.387
17	1.014	8.830	‘(s.cerevisiae)’ → ‘’	0.963	0.390
:	:	:	:	:	:
20	1.014	8.805	‘oncogene’ → ‘’	0.963	0.393
21	1.014	8.796	‘ingfinger’ → ‘nf’	0.963	0.394
22	1.014	8.790	‘isoformc’ → ‘’	0.963	0.395
23	1.014	8.783	‘ransmembrane’ → ‘mem’	0.963	0.395
24	1.014	8.778	‘ibosomal’ → ‘p’	0.964	0.396
25	1.014	8.770	‘subunit’ → ‘chain’	0.964	0.397
26	1.014	8.761	‘s.cerevisiaehomologof’ → ‘’	0.964	0.398
:	:	:	:	:	:
34	1.014	8.719	‘/’ → ‘f’	0.962	0.400
:	:	:	:	:	:
37	1.014	8.703	‘hypothetical’ → ‘’	0.962	0.402
:	:	:	:	:	:
41	1.014	8.685	‘eptid’ → ‘rote’	0.962	0.403
42	1.014	8.682	‘eucinerichrepeatcontaining’ → ‘rrc’	0.962	0.403
43	1.014	8.678	‘betadefensin’ → ‘defb’	0.962	0.404
:	:	:	:	:	:
57	1.014	8.639	‘molecule’ → ‘antigen’	0.962	0.405
:	:	:	:	:	:
62	1.014	8.631	‘oxonly’ → ‘x’	0.962	0.406
63	1.014	8.627	‘hromosome21openreadingframe’ → ‘21orf’	0.962	0.407
64	1.014	8.625	‘typeicytoskeletal’ → ‘’	0.962	0.408
:	:	:	:	:	:
68	1.014	8.611	‘member’ → ‘’	0.962	0.410
69	1.014	8.587	‘lfactoryreceptorfamily’ → ‘r’	0.963	0.413
:	:	:	:	:	:

The table clearly shows that the recall of lookup improved as we applied the discovered rules to the terms. More importantly, the degradation of precision was kept minimal. The discovered rules indicate that some technical words such as ‘protein’, ‘precursor’, ‘variant’ and ‘hypothetical’ are not important in conceptually characterizing a term and thus can be safely removed. The 14th rule is concerned with the acronym ‘il’. The rule effectively converts its long form ‘interleukin’ into the acronym. Some of the rules capture synonymous expressions. For example, the 25th rule replaces the word ‘subunit’ with ‘chain’.

Table 4 shows the result for the (reduced) disease dictionary. Again, the discovered rules improved the recall of lookup performance without causing a significant deterioration of precision. The rules discovered were very different from those for the gene/protein dictionary. For example, the fourth rule that removes ‘o’s enables us to match words in British spelling (e.g. ‘oesophageal’) with American counterparts (e.g. ‘esophageal’). The fifth rule that replaces ‘ies’ with ‘y’ can convert plural forms into singular. The 13th rule captures synonymous expressions in medical terminology (i.e. ‘kidniy’ (kidney) and ‘rinal’ (renal); note that ‘e’s are already converted to ‘i’s by a previous rule).

**Table 4:** Discovering rules from a disease dictionary

	Dictionary			Lookup performance	
Iter.	Ambiguity	Variability	Rule	Precision	Recall

0	1.001	2.794	*(convert capital letters to lower case)*	0.994	0.158
1	1.002	2.747	‘,’ → ‘’	0.989	0.184
2	1.002	2.667	‘ nos’ → ‘’	0.986	0.216
3	1.003	2.609	‘[x]’ → ‘’	0.985	0.263
4	1.003	2.580	‘o’ → ‘’	0.982	0.275
5	1.003	2.554	‘ies’ → ‘y’	0.983	0.291
6	1.003	2.529	‘ ’ → ‘-’	0.984	0.305
7	1.003	2.504	‘-’ → ‘;’	0.984	0.317
8	1.003	2.484	‘e’ → ‘i’	0.985	0.332
9	1.004	2.472	‘iasi’ → ‘rdir’	0.986	0.336
10	1.004	2.459	‘’s’ → ‘’	0.986	0.345
11	1.004	2.449	‘s’ → ‘z’	0.986	0.347
12	1.004	2.448	‘;(nz)’ → ‘’	0.986	0.347
13	1.004	2.447	‘kidniy’ → ‘rinal’	0.986	0.347
14	1.004	2.446	‘pulmnary’ → ‘lung’	0.986	0.347
15	1.004	2.443	‘ir’ → ‘ri’	0.986	0.348
16	1.004	2.441	‘aimia’ → ‘imiaz’	0.986	0.349
17	1.004	2.439	‘[d]’ → ‘’	0.986	0.349
18	1.004	2.436	‘aimlytic;animiaz’ → ‘imlytic;animia’	0.986	0.351
:	:	:	:	:	:
24	1.004	2.427	‘z;thi’ → ‘’	0.986	0.354
:	:	:	:	:	:
31	1.004	2.420	‘z;’ → ‘/’	0.986	0.355
32	1.004	2.348	‘/’ → ‘;’	0.987	0.377
33	1.004	2.348	‘dizrdri;liv’ → ‘livri;dizrd’	0.987	0.377
:	:	:	:	:	:
38	1.004	2.345	‘uding’ → ‘’	0.987	0.378
:	:	:	:	:	:
42	1.005	2.343	‘zufficiincy’ → ‘cmpitinci’	0.987	0.380
:	:	:	:	:	:
50	1.005	2.339	‘(in;zputum)’ → ‘in;zputum’	0.987	0.381
:	:	:	:	:	:
57	1.005	2.335	‘iincy’ → ‘’	0.987	0.382
:	:	:	:	:	:
70	1.005	2.333	‘[idta]’ → ‘’	0.987	0.385
:	:	:	:	:	:
89	1.005	2.327	‘ph’ → ‘f’	0.987	0.387
:	:	:	:	:	:
93	1.005	2.325	‘ci’ → ‘x’	0.987	0.388
:	:	:	:	:	:

### Evaluation using MEDLINE snippets

In the experiments presented in the previous section, we have demonstrated that the normalization rules discovered by our algorithm work well for unseen terms as well. It is, however, still not entirely clear how useful and safe those rules are. Although we used heldout data for evaluation, the nature of the heldout terms might be too similar to the remaining terms in the dictionary and thus we cannot rule out the possibility that the rules were actually overfitting the data. Moreover, the distribution of the terms in the dictionary is different from that of the terms appearing in real text, so the rules that are harmless within the dictionary might cause a problem of ambiguity when applied to terms in text.

To confirm the effectiveness of our normalization method, we need evaluation data that stem from real text rather than a dictionary. Fortunately, the BioCreAtIvE II gene normalization task [24] provides data which can be used for our experiments. The data (the “training.genelist” file) includes gene/protein name snippets extracted from MEDLINE abstracts, and each snippet is assigned an EntrezGene ID. Table 5 shows some examples of the snippets. This evaluation setting could be seen as the situation where we have a named entity recognizer that can *perfectly* identify the regions of gene/protein names in text. We converted the EntrezGene IDs to UniProt IDs so that they can be compared to the IDs in our human gene/protein dictionary. The resulting evaluation data consisted of 965 gene/protein name snippets and their IDs (there were 33 EntrezGene IDs that we failed to convert to UniProt IDs).

**Table 5:** Gene/protein name snippets. Examples of the gene/protein name snippets used in the lookup experiments reported in Table 6 and 7. The snippets are indicated in boldface type.

Snippets in context	EntrezGene IDs
… conserved in **VH1** and the **VH1-related (VHR) human protein**.	1845
These properties suggest that **VHR** is capable of regulating intracellular …	1845
… the kinase domain of the **keratinocyte growth factor receptor** ( …	2263
… (**bek/fibroblast growth factor receptor 2**) were infected with …	2263
The **Ah (dioxin) receptor** binds a number of widely disseminated …	196
… as a component of the DNA binding form of the **Ah receptor**.	196
:	:

With this evaluation data, we ran experiments using our gene/protein name dictionary (not the reduced version). The result is shown in Table 6. Again, the discovered rules improved the recall of lookup performance without losing precision. The main reason why the improvement of recall is not as significant as in Table 3,4 is that, unlike heldout terms, many of the snippets are readily mappable to the terms in the dictionary without any normalization. The useful rules were slightly different from the ones in Table 3. For example, the 38th rule, in effect, converts ‘receptor’ to ‘r’. The 44th rule converts ‘alpha’ to ‘a’. The Roman numeral ‘i’ is converted to the Arabic counterpart ‘1’ by the 75th rule.

**Table 6:** Evaluation using gene/protein name snippets from MEDLINE abstracts

	Dictionary			Lookup performance	
Iter.	Ambiguity	Variability	Rule	Precision	Recall

0	5.797	12.479	*(convert capital letters to lower case)*	0.782	0.582
1	5.807	12.161	‘-’ → ‘’	0.766	0.603
2	5.811	12.025	‘ precursor’ → ‘’	0.767	0.611
3	5.812	11.941	‘,’ → ‘’	0.767	0.611
4	5.812	11.907	‘inc finger protein’ → ‘nf’	0.767	0.611
5	5.812	11.868	‘ isoform 1’ → ‘’	0.767	0.611
6	5.813	11.832	‘ isoform 2’ → ‘’	0.766	0.611
7	5.813	11.806	‘ isoform a’ → ‘’	0.766	0.611
8	5.813	11.781	‘ isoform b’ → ‘’	0.766	0.611
9	5.813	11.748	‘ containing protein’ → ‘containing’	0.766	0.611
10	5.813	11.730	‘ variant’ → ‘’	0.766	0.611
:	:	:	:	:	:
21	5.815	11.597	‘nterleukin’ → ‘l’	0.767	0.613
:	:	:	:	:	:
24	5.816	11.566	‘specific’ → ‘’	0.767	0.615
:	:	:	:	:	:
33	5.816	11.450	‘protein’ → ‘gene’	0.765	0.616
34	5.828	11.056	‘ gene’ → ‘’	0.765	0.619
:	:	:	:	:	:
38	5.829	11.016	‘ recepto’ → ‘’	0.767	0.623
:	:	:	:	:	:
44	5.830	10.970	‘ alph’ → ‘’	0.765	0.625
:	:	:	:	:	:
75	5.831	10.838	‘ i’ → ‘1’	0.766	0.626
:	:	:	:	:	:
84	5.831	10.790	‘ lpha’ → ‘’	0.766	0.627
:	:	:	:	:	:
86	5.831	10.782	‘ beta’ → ‘b’	0.767	0.630
:	:	:	:	:	:
100	5.832	10.732	‘ type’ → ‘’	0.767	0.633

### Lookup performance

The greatest advantage of the normalization approach is the speed of looking up a dictionary. Once we normalize the terms in the dictionary and the input term, we can use a *hashing* technique to look it up in a constant time regardless of the dictionary size. The cost required for normalizing the terms in the dictionary is not a problem since it is done prior to processing the text. In contrast, the cost required for normalizing the input term could be an issue because we need to invoke the normalization process every time we come across a term in the course of text processing.

To see the computational overhead of normalization, we carried out experiments using the same dictionary and evaluation data used in the above experiment. We implemented the methods in C++ and ran the experiments on AMD Opteron 2.2GHz servers.

Table 7 shows the result. The bottom row shows the result of our automatic normalization method in which we used the 100 normalization rules discovered by the algorithm. We can see that the application of 100 rules made the lookup process several times slower than the case without any normalization. Note, however, that it is still more than ten thousand times faster than the soft matching cases where a simple character-level bigram similarity was employed. 0.67 seconds per lookup with soft matching may not appear to be hugely problematic, but it is not a desirable speed when we want to process a large amount of text or when real time processing is required (recall that we used only the human gene/protein dictionary in this experiment, which is a tiny fraction of the biomedical terminology).

**Table 7:** Dictionary lookup performance. This table shows the speed and accuracy of dictionary lookup tasks using the human gene/protein dictionary and gene/protein name snippets. F-score is the harmonic mean of precision and recall. The values in the parentheses are the threshold values in soft string matching.

Method	Precision	Recall	F-score	Average lookup time (microsecond)
Bigram similariy (0.97)	0.758	0.587	0.661	6.7 × 10^5^
Bigram similariy (0.95)	0.691	0.592	0.638	6.8 × 10^5^
Bigram similariy (0.93)	0.612	0.610	0.611	6.8 × 10^5^
No normalization	0.809	0.502	0.619	7
Case normalization	0.782	0.582	0.666	8
Heuristic normalization [18]	0.730	0.657	0.692	8
**Automatic normalization**	0.767	0.633	0.694	29

We should nevertheless emphasize that the purpose of this work is not to claim that our automatic term normalization approach is superior to soft string matching approaches. Soft matching methods have a distinct advantage of being able to output similarity scores for matched terms. Also, soft matching is in general more robust to various transformations than normalization approaches. The heavy computational cost is not a problem in certain applications. Soft matching and normalization are, in fact, complementary.

The table also shows the performance achieved by the heuristic rules given in Fang et al. [18]. The normalization consists of case normalization, replacement of hyphens with spaces, removal of punctuation, removal of parenthesized materials, and removal of spaces. Their normalization gave a better recall than our system at the price of a degradation of precision. Among their normalization rules, removal of parenthesized materials is particularly interesting, because this rule can never be produced by our algorithm. This is an instance of “clever” rules that are difficult to discover without the help of human knowledge.

We conducted a brief error analysis on the results of this mapping task to see what types of term variations were yet to be captured by the system. Somewhat surprisingly, there were still many terms that could be mappable via character-level replacement rules. This indicates that we could improve the rule discovery process by employing a more sophisticated method to explore the hypothesis space. Our rule discovery algorithm has some commonalities with Transformation Based Learning (TBL) [25], so the approaches proposed to improve the training process in TBL (e.g. [26] and [27]) may also be useful in pursuing this direction. The other types of unresolved variations include different word ordering (e.g. ‘IgA Fc receptor’ and ‘Fc receptor for IgA’) and coordination (e.g. ‘ZNF133, 136 and 140’).

## Conclusions

Developing good heuristics for term normalization requires extensive knowledge of the terminology in question, and it is the bottleneck of normalization approaches for term-concept mapping tasks. In this paper, we have shown that the automatic development of normalization rules is a viable solution to the problem, by presenting an algorithm that can discover effective normalization rules from a dictionary. The algorithm is easy to implement and efficient enough that it is applicable to large dictionaries. Experimental results using a human gene/protein dictionary and a disease dictionary have shown that the automatically discovered rules can improve recall without a significant loss of precision in term-concept mapping tasks. This work should be particularly useful for terminologies for which good normalization rules are not fully known.

In this work, we limited the type of normalization rules to character-level replacement. There are, however, many good heuristics that cannot be captured in this framework. Extending the scope of normalization rules to more flexible expressions is certainly an interesting direction of future work.

## Competing interests

The authors declare that they have no competing interests.

## Authors' contributions

YT developed the algorithm, carried out the experiments and drafted the manuscript. JM and SA conceived the study and participated in its design and coordination. All authors read and approved the final manuscript.
